# Efficacy of plant extracts for metabolic dysfunction-associated steatotic liver disease: a network meta-analysis

**DOI:** 10.3389/fphar.2026.1919204

**Published:** 2026-09-29

**Authors:** Zhenghong Xie, Tianxing Chen

**Affiliations:** Medical School, Nantong University, Nantong, Jiangsu, China

**Keywords:** liver enzyme, metabolic dysfunction-associated steatotic liver disease, network meta-analysis, Phytotherapy, plant extract, randomized controlled trial

## Abstract

**Background:**

Metabolic dysfunction-associated steatotic liver disease (MASLD) is a leading cause of chronic liver disease worldwide, affecting approximately 38% of the global population. This network meta-analysis systematically compared the effects of different plant extracts on liver biochemical and non-invasive histological outcomes in patients with MASLD.

**Methods:**

This study was performed in accordance with the PRISMA guidelines and registered with PROSPERO (CRD420251229711). We systematically searched PubMed, Embase, Cochrane Library, and Web of Science up to November 12, 2025 for randomized controlled trials (RCTs) assessing plant extracts in adults with MASLD. Primary outcomes were alanine aminotransferase (ALT), aspartate aminotransferase (AST), controlled attenuation parameter (CAP), liver stiffness measurement (LSM), and hepatic steatosis percentage. Network meta-analyses were conducted using Stata 15.1 and R 4.2.1.

**Results:**

In total, 32 RCTs were included, involving 2,053 patients and 28 types of plant extracts. For liver enzyme outcomes, a novel curcumin preparation (CP) exhibited the highest ranking probability for ALT reduction (SUCRA: 91.93%), whereas cinnamon (Cin) ranked highest for AST reduction (SUCRA: 88.48%). For non-invasive histological outcomes, purslane (Pur) showed the greatest ranking probability for reducing LSM (SUCRA: 94.16%), while fenugreek (Fen) demonstrated favorable ranking probabilities for both CAP reduction (SUCRA: 79.07%) and hepatic steatosis percentage reduction (SUCRA: 86.97%). The overall methodological quality of the included studies was generally acceptable, and funnel plot assessments revealed no substantial evidence of publication bias.

**Conclusion:**

Compared with placebo, most plant extracts do not achieve statistically significant improvements in liver biochemical or non-invasive liver-related outcomes, leaving their overall clinical efficacy uncertain. Although CP, Cin, Fen, and Pur showed favorable probability rankings for specific endpoints based on SUCRA values, these findings should not be interpreted as evidence of clinically meaningful superiority. Given the limited evidence, considerable heterogeneity, and uncertainty in comparative estimates, the current data do not support the use of plant extracts as standalone therapies for MASLD outside the context of concomitant lifestyle modification. Adequately powered, standardized RCTs are warranted to clarify their therapeutic effects.

**Systematic Review Registration:**

https://www.crd.york.ac.uk/PROSPERO/view/CRD420251229711; Identifier: CRD420251229711.

## 1 Introduction

Metabolic dysfunction-associated steatotic liver disease (MASLD) (Rinella et al., 2023), previously termed nonalcoholic fatty liver disease (NAFLD), has emerged as a major global health burden, with an estimated prevalence rate of 38% among adults worldwide and a projected increase to 55.4% by 2040 (Younossi et al., 2023; Le et al., 2022). As a systemic metabolic disorder, MASLD is associated with progressive hepatic damage and heightened risks of cardiovascular and metabolic complications (Armstrong et al., 2014), underscoring the urgent need for effective early interventions. Current clinical management strategies for MASLD primarily encompass lifestyle intervention, pharmacotherapy, and bariatric surgery. To date, however, no globally approved specific drug therapy has been established. According to prevailing international guidelines, lifestyle modification (particularly dietary optimization combined with regular physical activity) remains the cornerstone of MASLD care. Clinical evidence indicates that a 5%–10% reduction in body weight significantly ameliorates hepatic steatosis and lowers serum aminotransferase levels (Tacke et al., 2024). Nevertheless, the long-term sustainability of lifestyle interventions is often hampered by poor patient adherence and difficulties in maintaining behavioral changes over time (Shibayama et al., 2024). Although pharmacological and surgical options may provide additional benefits for selected patients, their broader clinical application is constrained by potential adverse effects, high costs, and limited efficacy in reversing advanced fibrosis (Sanyal et al., 2025; Harrison et al., 2024; Lassailly et al., 2020). Therefore, there remains a pressing need for safer, better-tolerated, and more widely applicable adjunctive therapies for MASLD. In this context, natural products and herbal medicines have garnered growing attention owing to their potential multi-target, multi-pathway regulatory properties (Yao et al., 2016). Unlike conventional single-target agents, plant-derived compounds may simultaneously modulate multiple pathological processes implicated in MASLD progression, including oxidative stress, inflammation, and lipid metabolism disturbances (Scapagnini et al., 2011; Jomova et al., 2025).

Over recent years, an increasing number of randomized controlled trials (RCTs) have investigated the potential of specific plant extracts in optimizing MASLD-related outcomes. Although the effects of natural products in MASLD have been explored in previous studies, existing evidence syntheses have focused primarily on particular intervention categories or a restricted set of outcome indicators. Methodologically, Yang et al. (2022) employed conventional meta-analysis to show that dietary polyphenols may be beneficial for MASLD-related indicators, providing foundational support for the potential of bioactive compounds. However, conventional meta-analysis does not directly compare or rank multiple interventions, and therefore, the relative effects of different plant-derived interventions remain to be further clarified. Subsequently, Liu et al. (2024) performed a network meta-analysis (NMA) comparing several natural products, including curcumin and silymarin, in individuals with MASLD, providing valuable insights into the comparative effects of selected traditional botanicals. Nevertheless, as evidence from emerging plant extracts continues to accumulate, the comparative efficacy of a broader range of plant-derived interventions has not yet been fully characterized within an NMA framework. More recently, Sairaj et al. (2026) have further expanded the understanding of herbal products in MASLD by providing additional evidence on metabolic parameters (Sairaj et al., 2026). However, for plant extracts particularly, the comparative effects on liver-related outcomes across different biochemical markers and non-invasive surrogates remain to be further explored.

Against this backdrop, this study, through an NMA incorporating recent RCTs, intended to systematically compare the relative efficacy of diverse plant extracts in MASLD. By integrating conventional biochemical indicators with non-invasive liver-related surrogate outcomes, this study aimed to provide complementary evidence on the potential role of different plant extracts as adjunctive interventions for MASLD management, thereby extending and refining the existing evidence base.

## 2 Methods

This study was performed in accordance with the Preferred Reporting Items for Systematic Reviews and Meta-Analyses (PRISMA) reporting guideline (Hutton et al., 2015) and its requirements for NMAs (Supplementary Appendix 1). The study protocol was registered with the International Prospective Register of Systematic Reviews (PROSPERO: CRD420251229711).

### 2.1 Literature search

PubMed, Embase, Cochrane Library, and Web of Science were systematically searched from database inception to November 12, 2025. The search strategy combined Medical Subject Headings (MeSH) and free-text terms, with MeSH terms covering MASLD, NAFLD, and related disease terminology, and the intervention term ‘extract’ for initial screening. To enhance the recall rate and ensure the inclusion of randomized studies, additional design-related terms such as ‘clinical trial’, ‘trial’, and ‘blind’ were further added, in addition to the original search terms including ‘random’, ‘RCT’, and ‘placebo’. Detailed search strategies are available in Supplementary Appendix 2. Additionally, a supplementary secondary search was performed by reviewing the reference lists of published systematic reviews to ensure comprehensive literature coverage.

### 2.2 Eligibility criteria

Studies meeting the following criteria were included.

- Participant: Adults (≥18 years old) with a diagnosis of MASLD were eligible.
- Intervention: A total of 28 types of plant extracts were included as interventions. Patients in an intervention group received single-component extracts (e.g., silymarin, curcumin preparation [CP], green tea, and resveratrol), medicine-food homology extracts (e.g., cinnamon [Cin], purslane [Pur], and red ginseng), or compound proprietary preparations (e.g., a compound of bergamot polyphenol fraction [BPF], fucoxanthin, and pomegranate seed oil). No restrictions were imposed on daily diet or physical activity during the intervention. A control group received a placebo, specifically including simulated capsules composed of starch, wheat flour, or other substances without pharmacological activity. Restrictions on diet or exercise in a control group were kept consistent with those in an intervention group.
- Study design: Only RCTs were eligible.
- Outcome indicator: Studies reporting at least one of the following outcomes were included: alanine aminotransferase (ALT), aspartate aminotransferase (AST), controlled attenuation parameter (CAP), liver stiffness measurement (LSM), and hepatic steatosis percentage.

Studies were excluded if they were: (i) animal or cell experiments, case reports, scientific experimental protocols, comments, letters, editorials, or conference papers; (ii) studies with insufficient data for quantitative synthesis due to incomplete reporting or unresolvable errors; (iii) duplicate publications (only the study with the most complete data was retained); or (iv) studies for which a full text was not available even after an attempt to contact the original authors.

### 2.3 Literature screening and data extraction

All retrieved records were imported into EndNote 21. Two researchers independently assessed all titles and abstracts against the eligibility criteria, followed by full-text assessment. Any disagreements were reconciled through discussion or by consulting a third researcher. Using a pre-designed data extraction form, the two researchers independently extracted data from all included studies: first author, publication year, country, intervention and control measures, treatment duration, dosage, baseline characteristics of participants, and outcome indicators.

### 2.4 Assessment of transitivity assumption

Before NMA, the transitivity assumption was assessed by descriptively comparing potential effect modifiers across the included studies. The evaluated factors were participant age, baseline liver enzyme levels, treatment duration, and intervention dosage. Similarity in the distribution of these characteristics among studies was considered supportive of the plausibility of the transitivity assumption.

### 2.5 Risk of bias assessment

The Cochrane RoB 2.0 (Sterne et al., 2019) was used to assess the risk of bias in each included study across five domains: randomization process, deviations from intended interventions, missing outcome data, measurement of outcomes, and selection of reported results. Two reviewers independently rated each domain as having ‘a low risk’, ‘a high risk’, or ‘some concerns’. Any disagreements were reconciled via discussion or by consulting a third researcher. Assessment results were detailed in a risk-of-bias figure.

### 2.6 Statistical analysis

All statistical analyses were performed using Stata 15.1 and R 4.2.1. All outcomes were continuous variables, and the effect size was expressed as the weighted mean difference (WMD) with 95% credible interval (CrI). The transitivity assumption underpins valid indirect comparisons in NMA. In this study, we assessed this assumption qualitatively by constructing a summary table of key study-level characteristics: age, sex, body mass index (BMI), baseline ALT/AST levels, treatment duration, and intervention dosage, to evaluate the comparability of potential effect modifiers across different interventions. Given the potential heterogeneity across studies, a Bayesian hierarchical random-effects model was first fitted to perform multiple comparisons among different interventions for MASLD (Dias et al., 2013; Mills et al., 2013). Based on the likelihood function and pre-specified prior distributions, Markov chain Monte Carlo (MCMC) simulations were executed in R 4.2.1 to obtain posterior distributions of the model parameters (Dias et al., 2012; Bois, 2013; Hamra et al., 2013). The number of model iterations was set to 50,000, with the first 20,000 discarded as burn-in to eliminate the influence of initial values. The stability of the trace density plots and the Brooks-Gelman-Rubin diagnostic plot was examined to assess model convergence (PSRF <1.1). For model selection, a consistency model was compared with an inconsistency model using the Deviance Information Criterion (DIC). If the absolute DIC difference between the two models was below 5, global consistency was considered acceptable, and the more parsimonious consistency model was preferentially used (Dias et al., 2013; Spiegelhalter et al., 2002). If substantial inconsistency was detected, a node-splitting method was applied to identify inconsistent nodes. Heterogeneity was assessed using the I^2^ statistic, with values of 25%, 50%, and 75% considered as low, moderate, and high heterogeneity, respectively. To examine whether treatment effects were influenced by clinical characteristics, we performed univariable random-effects network meta-regression analyses based on treatment duration and plant extract dosage, as well as subgroup analyses stratified by the presence of structured lifestyle interventions and by plant source. Network plots were generated to visualize the relationships among interventions. The relative efficacy of each intervention was ranked based on surface under the cumulative ranking curve (SUCRA) values. A SUCRA value ranges from 0 to 1, with a larger value indicating that the corresponding intervention is more effective in treating MASLD (Rücker and Schwarzer, 2015; Trinquart et al., 2016), though this does not equate to real-world clinical benefit. Pairwise comparisons between interventions for each outcome indicator were presented in league tables. Potential publication bias was assessed through comparison-adjusted funnel plots (Chaimani et al., 2013; Whegang et al., 2017).

### 2.7 Confidence in network meta-analysis (CINeMA) assessment

Two researchers independently assessed the confidence in the evidence for each outcome using the CINeMA framework. This approach evaluates six domains: within-study bias, across-study bias, indirectness, imprecision, heterogeneity, and incoherence. The assessment followed the methodological standards outlined by Nikolakopoulou et al., with all judgments performed via the CINeMA web application (CINeMA, 2020; Nikolakopoulou et al., 2020; Papakonstantinou et al., 2020). Any discrepancies between the two researchers were reconciled through discussion.

## 3 Results

### 3.1 Literature search and screening

In total, 3,674 records were initially retrieved. After the removal of 1,406 duplicate records, 2,155 records were excluded based on an assessment of titles and abstracts. Full texts could not be obtained for 7 studies, leaving 106 articles for full-text assessment. At this stage, 74 studies were excluded: 35 did not align with the study objective, and 39 lacked relevant or valid data for quantitative synthesis. Ultimately, 32 studies were included. A literature screening is detailed in Figure 1.

**FIGURE 1:**
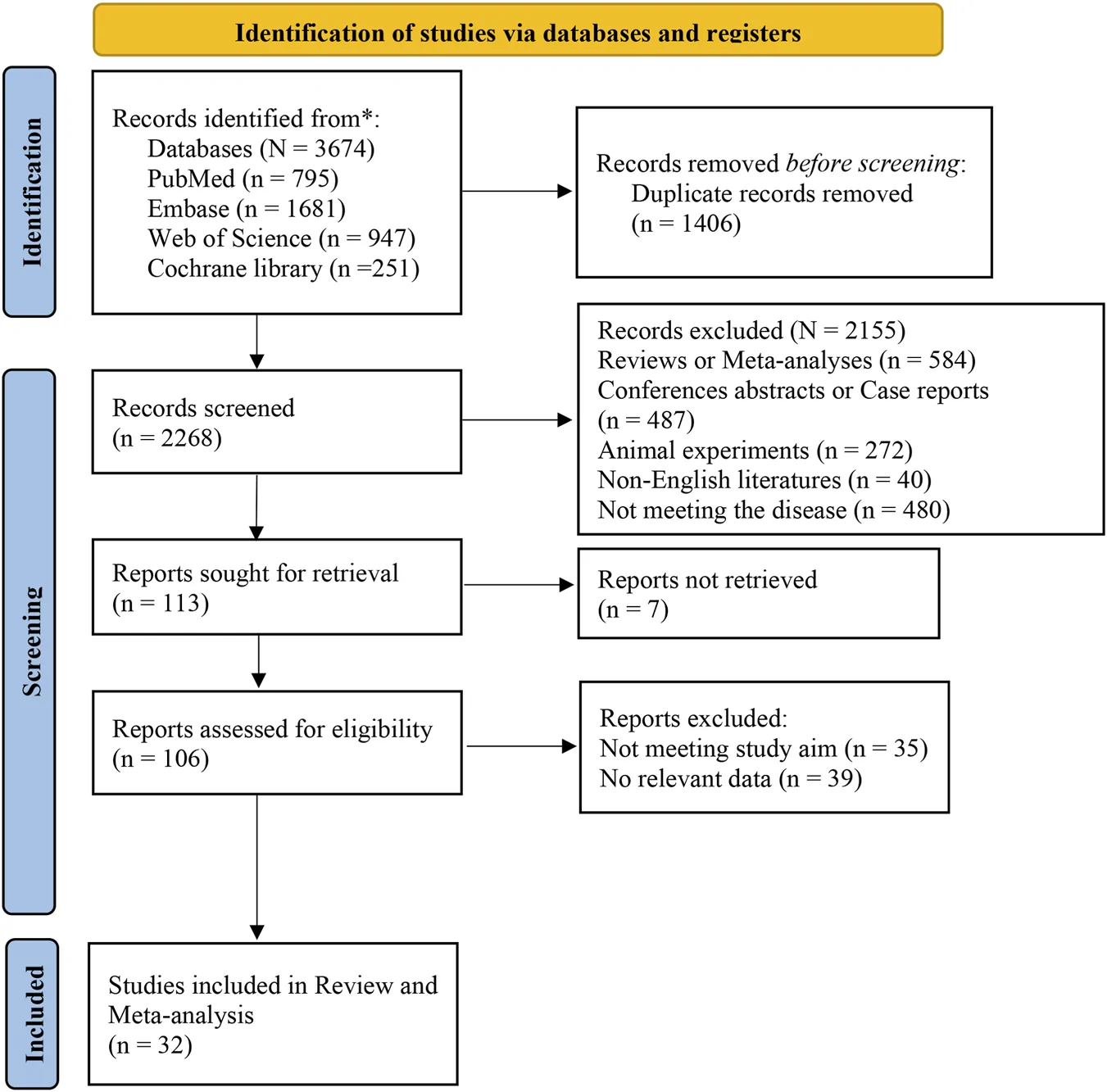
PRISMA flow diagram.

### 3.2 Characteristics of included studies

A total of 32 RCTs (Ghanbari et al., 2024; Musolino et al., 2020; Nikkhajoei et al., 2016; Patel et al., 2025; Mirhashemi et al., 2022; Rajendra et al., 2022; Abidov et al., 2010; Abu Hassan et al., 2023; Shahmohammadi et al., 2017; Pezeshki et al., 2016; Askari et al., 2014; Chang et al., 2014; Chayanupatkul et al., 2022; Sangsefidi et al., 2021; Hussain et al., 2017; Ferro et al., 2020; Han et al., 2020; Maurotti et al., 2024; Hosseinab et al., 2019; Ferro et al., 2022; Milkarizi et al., 2024; Parsi et al., 2020; Faghihzadeh et al., 2015; Hong et al., 2021; Jafa et al., 2023; Zamani et al., 2018; Izadi et al., 2021; Jinato et al., 2022; Lewis et al., 2018; Babaei et al., 2020; Faghihzadeh et al., 2014; Iloon Kashkooli et al., 2015) involving 2,053 participants were included. These studies originated from 10 countries and regions: Iran (17 studies), Italy (4 studies), India (2 studies), South Korea (2 studies), Thailand (2 studies), the United States (1 study), Russia (1 study), Malaysia (1 study), Taiwan (China) (1 study), and Pakistan (1 study). These studies involved 28 types of plant extracts or compound preparations, including silymarin, green tea, resveratrol, curcumin, and BPF. Sample sizes ranged from 24 to 226 participants, most of whom were adults aged 25–52 years. Intervention durations varied between 4 weeks and 12 months. All studies reported ALT and AST levels, and some studies further assessed LSM, CAP, and hepatic steatosis percentage. The characteristics of all included studies are detailed in Table 1.

**TABLE 1:** Baseline characteristics of included studies.

First author	Publication year	Country	Group	Sample size	Sex (M/F)	Age	Intervention dose(mg/d)	Treatment duration(w)	Outcomes
Babaei A et al.	2020	Iran	Fenugreek	13	13/0	39.08 ± 11.00	1,000	12	①②③④⑤
			Placebo	11	9/2	39.55 ± 8.70			
Musolino V et al.	2020	Italy	BPF + CyC	20	NA	51 ± 8	600	16	①②
			BPF	20		51.0 ± 9			
			CyC	20		50 ± 9			
			Placebo	20		51 ± 10			
Nikkhajoei M et al.	2016	Iran	Chicory	31	27/4	41.2 ± 12.5	500	8	①②
			Placebo	30	24/6	41.8 ± 11.4			
Patel G	2023	India	Curcumin preparation	15	12/3	31.4 ± 6.08	500	8.6	①②⑤
			Placebo	15	12/3	36.9 ± 10.50			
Mirhashemi SH et al.	2022	Iran	Silymarin	27	NA	37.81 ± 9.93	560	8	①②
			Placebo	25		38.08 ± 10.01			
Pothula Rajendra VK et al.	2022	India	CL16049F1	30	14/16	36.9 ± 9.2	300	12	①②
			Silymarin	30	16/14	40.2 ± 10.1			①②
			Placebo	30	16/14	36.6 ± 7.5			①②
Abidov M et al.	2010	Russia	Xanthigen	36	NA	37.4 ± 2.8	600	16	①②④
			Placebo	36		36.1 ± 2.1			
Abu Hassan MR et al.	2023	Malaysia	Phyllanthus niruri	112	56/56	50.71 ± 12.26	3,000	48	①②⑤
			Placebo	114	51/63	49.80 ± 11.93			
Shahmohammadi HA et al.	2017	Iran	GCBE	22	11/11	41.36 ± 7.69	1,000	8	①②
			Placebo	22	11/11	44.50 ± 5.24			
Alavinejad P	2019	Iran	Green tea extract	35	18/17	NA	500	12.9	①②
			Placebo	36	21/15				
Askari F	2013	Iran	Cinnamon	23	10/13	44.8 ± 8.5	1,500	12	①②
			Placebo	22	11/11	45.4 ± 8.2			
Chang HC et al.	2014	Taiwan	Hibiscus sabdariffa extract	19	12/7	37.3 ± 8.6	3,000	12	①②
			Placebo	17	9/8	38.6 ± 10.5			
Chayanupatkul M	2022	Thailand	Oligonol	20	12/8	50.6 ± 12.0	200	24	①④
			Placebo	20					
Sangsefidi et al.	2021	Iran	CMFE	25	12/13	41.48 ± 9.53	2,800	48	①②
			Placebo	25	11/14	42.68 ± 9.96			
Hussain M et al.	2017	Pakistan	Green tea extract	40	26/14	25 ± 18	1,000	12	①②
			Placebo	40	28/12	28 ± 15			
Faghihzadeh F	2014	Iran	TR	25	18/7	44.04 ± 10.10	500	12	①②
			Placebo	25	17/8	46.28 ± 9.52			
Han B et al.	2020	Korea	Artemisia annua L	38	26/12	48.00 ± 12.63	1,372	8	①②
			Placebo	41	32/9	49.20 ± 12.94			
Ferro Y et al.	2020	Italy	BC	51	28/23	51 ± 9	300	12	①②③⑤
			Placebo	51	29/22				
Maurotti Samantha et al.	2023	Italy	BPF + CyC	16	11/5	51 ± 8	300	12	①②⑤
			Placebo	16	11/5	52 ± 11			
Hosseinabadi S	2019	Iran	Green coffee extract	21	11/10	41.14 ± 7.87	400	8	①②
			Placebo	23	12/11	41.13 ± 8.47			
Iloon Kashkooli R et al.	2015	Iran	Berberis vulgaris	40	19/21	43.65	1,500	12	①②
			Placebo	40	13/27	42.97			
Ferro Y et al.	2022	Italy	BPF + CyC	47	28/19	50–52	300	6	①②
			Placebo	47	26/21	50–52			
Milkarizi N et al.	2024	Iran	Purslane	35	18/17	43.8 ± 7.6	700	8	①②③
			Placebo	35	18/17	44.2 ± 9.6			
Ghanbari et al.	2024	Iran	Grape seed extract	23	10/13	42.13 ± 8.45	520	8	①②
			Placebo	23	9/14	41.69 ± 7.42			
Faghihzadeh F et al.	2015	Iran	Resveratrol	25	18/7	44.04 ± 10.10	500	12	①②
			Placebo	25	17/8	46.28 ± 9.52			
Paris A et al.	2019	Iran	Crocus sativus	30	16/14	36.1 ± 45.47	15	8	①②
			Placebo	30	17/13	33.08 ± 2.8			
Hong JT et al.	2020	Korea	Korea red ginseng	48	27/21	50.0 ± 13.3	2000	4	①②
			Placebo	46	29/17	49.7 ± 13.2			
Jafarirad S et al.	2022	Iran	Pomegranate	22	NA	46 ± 9.4	450	12	①②
			Placebo	22					
Zamani N	2018	Iran	Zataria multiflora Boiss	40	25/15	36.8 ± 9.2	700	12	①②
			Placebo	45	36/9	41 ± 11			
Izadi F	2020	Iran	Sour tea	30	17/13	43.3 ± 10.2	450	8	①②
			Placebo	31	19/12	42.8 ± 10.6			
Lewis JE et al.	2015	USA	Rice Bran Arabinoxylan compound	12	5/7	58.1 ± 15.2	1,000	12	①②
			Placebo	11	6/5	50.6 ± 11.6			
Jinato T	2022	Thailand	Oligonol	19	12/7	50.8 ± 10.1	200	24	①②
			Placebo	19	11/8	49.5 ± 13.8			

①: Aspartate Transferase (AST); ②: Alanine Aminotransferase (ALT); ③: Liver Stiffness Measurement (LSM); ④: Steatosis percent; ⑤: Controlled Attenuation Parameter (CAP).

BPF, bergamot polyphenolic fraction; CyC cynara cardunculus; BC, bergamot citrus and wild cardoon; CFP, Capparis spinosa.

CL16049F1 A proprietary blend of *Sphaeranthus indicus* flower head and Terminalia chebula fruit extracts; GCBE, green coffee bean extract; TR: trans-resveratrol CMFE: Cornus mas L. fruit extract.

### 3.3 Assessment of transitivity assumption

The distribution of potential effect modifiers was evaluated across all included studies. The study populations were generally comparable in terms of age, baseline liver enzyme levels, and treatment duration. No marked imbalances in these characteristics were observed across direct comparisons, supporting the plausibility of the transitivity assumption (Supplementary Appendix 3).

### 3.4 Methodological quality assessment

Risk of bias was assessed using the Cochrane RoB 2.0 (Figure 2). Regarding randomization process, 24 studies were judged as having a low risk of bias, and 8 as having some concerns. Concerning deviations from intended interventions, 32 studies were rated as having a low risk of bias, and 1 as having some concerns. For missing outcome data, 27 studies were assessed as having a low risk of bias, 2 as having some concerns, and 3 as having a high risk of bias. All studies were rated as having a low risk of bias in measurement of outcomes. Regarding selection of reported results, 19 studies were judged as having a low risk of bias, 13 as having some concerns, and 1 as having a high risk of bias. Overall, 17 studies were of low risk, 12 had some concerns, and 3 were of high risk. Common concerns included inadequate reporting of randomization procedures, missing outcome data, and selective reporting. These methodological limitations might have introduced uncertainty into the estimated treatment effects and could influence the robustness of pooled estimates and treatment rankings, particularly for comparisons with limited evidence. Although measurement of outcomes was considered at low risk of bias due to the objective nature of biochemical and imaging assessments, potential concerns in other domains should be considered when interpreting the findings.

**FIGURE 2:**
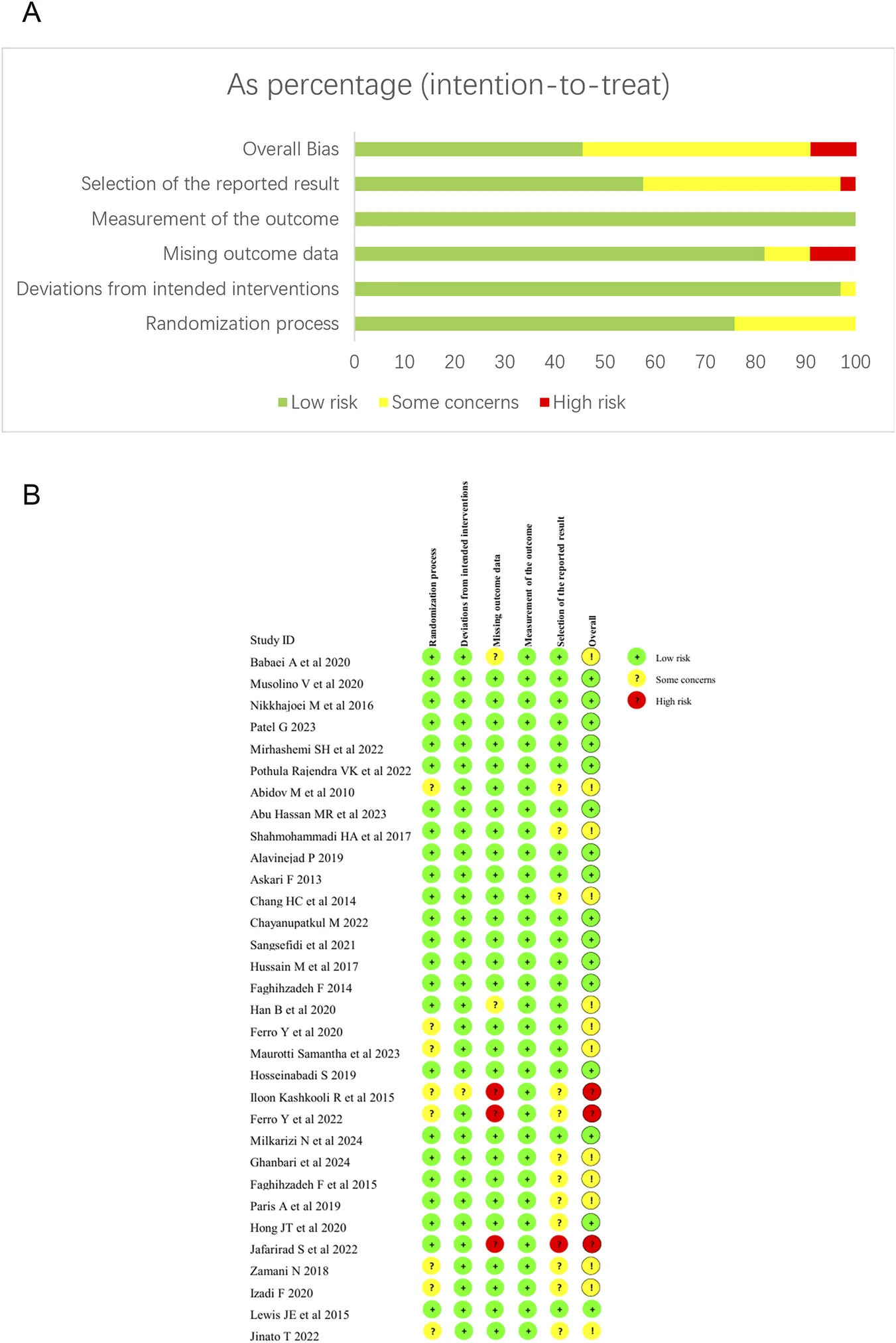
Risk of bias assessment. **(A)** Risk of bias summary across the included studies. **(B)** Risk of bias assessment for individual studies.

### 3.5 NMA

#### 3.5.1 Network plot

In total, 32 studies reported ALT, covering 28 types of plant extracts. AST was reported in 31 studies, with 28 categories of plant extracts involved. Data on CAP were provided in 5 studies, with 4 types of plant extracts covered. Three studies provided data on LSM, involving three categories of plant extracts. Hepatic steatosis percentage was reported in 3 studies, covering three types of plant extracts. Details are shown in Figures 3A–E. In the network plots, the thickness of a line is proportional to the number of direct pairwise comparisons, and the diameter of each circle is proportional to the number of participants receiving the corresponding intervention.

**FIGURE 3:**
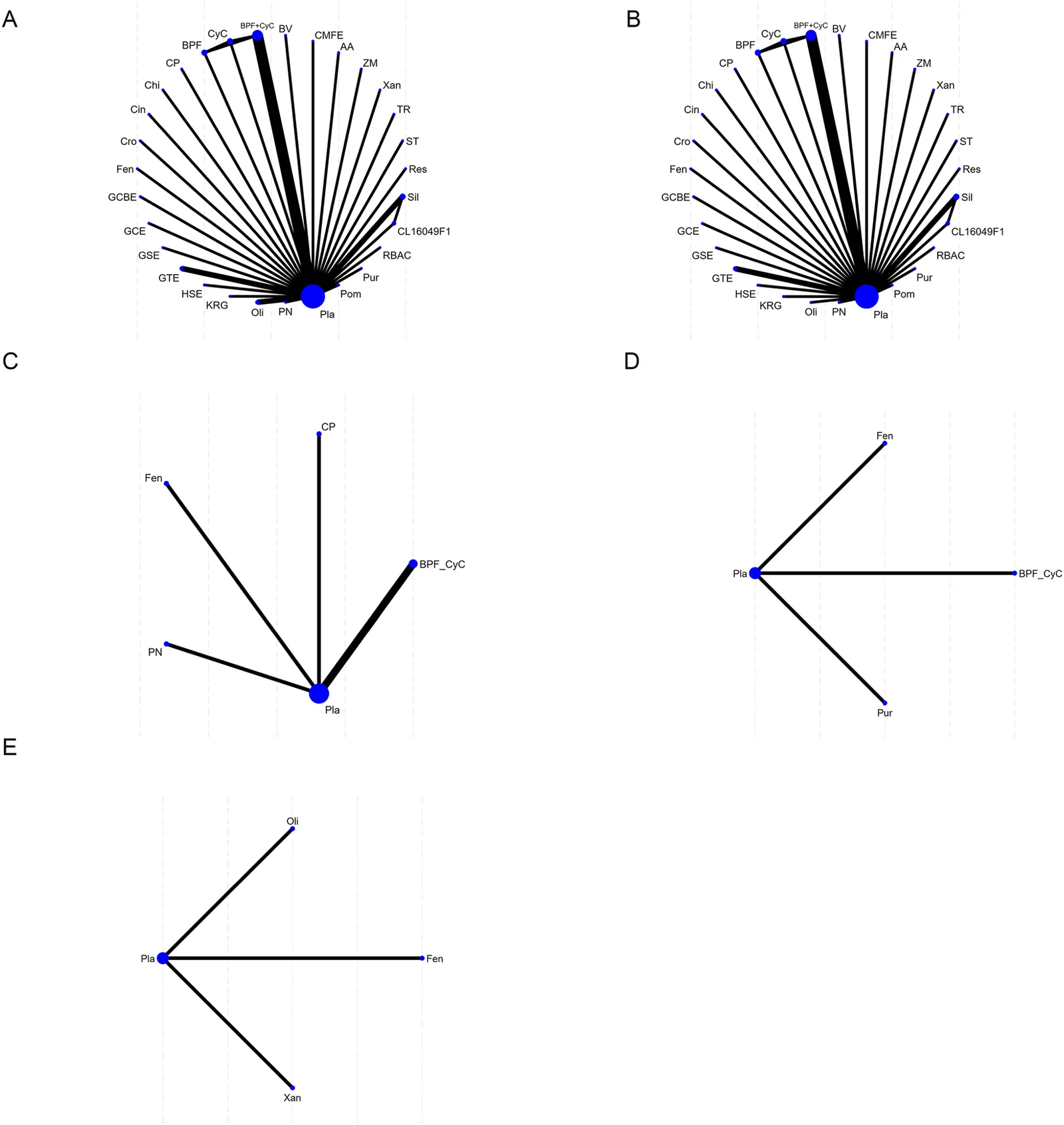
Network plot. **(A)** ALT. **(B)** AST. **(C)** CAP. **(D)** LSM. **(E)** Hepatic steatosis percentage.

#### 3.5.2 Consistency assessment

Consistency was assessed based on the DIC. The observed ΔDIC values were all below 5: ALT (ΔDIC = 0.15), AST (ΔDIC = 0.7), LSM (ΔDIC = 0.01), CAP (ΔDIC = 0.05), and hepatic steatosis percentage (ΔDIC = 0.02) (Supplementary Appendix 4). These results suggested that incorporating inconsistency parameters did not substantially improve model fit. Therefore, the consistency model was considered appropriate and was applied for all primary NMAs.

#### 3.5.3 ALT

The league table for ALT is presented in Table 2. In the pairwise comparisons of different types of plant extracts, CP was associated with a greater ALT reduction compared with BPF + *Cynara cardunculus* (CyC) (MD: −24.28, 95% CrI: −47.84 to −0.79), fenugreek (Fen) (MD: −43.28, 95% CrI: −81.96 to −4.67), *Hibiscus sabdariffa* extract (HSE) (MD: −34.25, 95% CrI: −67.93 to −0.75), Oligonol (Oli) (MD: −31.44, 95% CrI: −58.26 to −4.69), and placebo (MD: −29.25, 95% CrI: −50.43 to −8.14). Additionally, Cin was potentially more effective than Fen (MD: −39.21, 95% CrI: −77.19 to −1.09), Oli (MD: −27.25, 95% CrI: −53.53 to −1.04), and placebo (MD: −25.10, 95% CrI: −45.65 to −4.49) in reducing ALT levels. No statistically significant differences were observed among the remaining plant extracts (P > 0.05). According to the SUCRA ranking, CP (SUCRA: 91.93%), Cin (SUCRA: 87.60%), and *Crocus sativus* (Cro) (SUCRA: 78.00%) had a higher probability of being top-ranked for ALT reduction (Figure 4A and Supplementary Appendix 5).

**TABLE 2:** Network meta-analysis league table for ALT.

AA														
−1.96 (−29.06, 25.24)	BPF													
−0.68 (−23.62, 22.26)	1.32 (−16.73, 19.12)	BPF_CyC												
10.67 (−18.21, 39.52)	12.65 (−14.36, 39.64)	11.31 (−11.38, 34.16)	BV											
−3.23 (−37.28, 31.57)	−1.13 (−34.03, 31.75)	−2.48 (−32.01, 27.29)	−13.8 (−48.16, 20.59)	Chi										
19.42 (−9.62, 48.66)	21.44 (−6.06, 48.67)	20.1 (−2.99, 43.26)	8.78 (−20.18, 37.58)	22.63 (−12.05, 56.87)	Cin									
0.43 (−27.55, 28.18)	2.41 (−23.7, 28.45)	1.09 (−20.47, 22.68)	−10.23 (−38.05, 17.48)	3.57 (−30, 37)	−19.04 (−46.91, 8.82)	CL16049F1								
−6.4 (−38.28, 25.54)	−4.37 (−34.61, 25.88)	−5.68 (−32.41, 21.07)	−17.06 (−48.79, 14.81)	−3.24 (−40.45, 33.94)	−25.84 (−57.76, 6.25)	−6.79 (−37.78, 24.09)	CMFE							
23.59 (−5.57, 53.04)	25.6 (−2.17, 53.13)	24.28 (0.79, 47.84)	12.94 (−16.47, 42.19)	26.71 (−8.06, 61.72)	4.17 (−25.47, 33.78)	23.15 (−4.97, 51.81)	29.96 (−2.35, 62.43)	CP						
13.31 (−15.87, 42.56)	15.35 (−12.1, 42.7)	14.02 (−9.14, 37.17)	2.68 (−26.46, 31.58)	16.5 (−18.15, 51.11)	−6.06 (−35.26, 23.24)	12.88 (−15.19, 41.03)	19.71 (−12.3, 51.73)	−10.25 (−39.98, 19.33)	Cro					
1.01 (−26.21, 27.96)	2.97 (−16.8, 22.6)	1.67 (−16.17, 19.64)	−9.68 (−36.67, 17.16)	4.12 (−28.97, 36.89)	−18.44 (−45.89, 9.03)	0.55 (−25.35, 26.74)	7.37 (−22.95, 37.95)	−22.62 (−50.25, 4.98)	−12.35 (−39.52, 15.01)	CyC				
−19.82 (−57.62, 18.39)	−17.75 (−54.41, 19.02)	−19.08 (−52.98, 14.88)	−30.41 (−68.32, 7.56)	−16.54 (−59.17, 25.81)	−39.21 (−77.19, −1.09)	−20.18 (−57.33, 17.3)	−13.35 (−53.86, 27.18)	−43.28 (−81.96, −4.67)	−33.1 (−71.25, 5.31)	−20.76 (−57.41, 16.08)	Fen			
1.73 (−26.68, 30.29)	3.75 (−23.13, 30.51)	2.41 (−19.95, 25.01)	−8.95 (−37.21, 19.41)	4.9 (−29.26, 38.87)	−17.68 (−46.27, 10.99)	1.29 (−25.92, 28.74)	8.15 (−23.49, 39.65)	−21.84 (−50.9, 7.22)	−11.58 (−40.2, 17.08)	0.74 (−25.93, 27.53)	21.48 (−16.25, 59.21)	GCBE		
−5.75 (−37.28, 26.19)	−3.71 (−33.67, 26.36)	−5.07 (−31.23, 21.46)	−16.42 (−47.68, 15.14)	−2.53 (−39.55, 34.25)	−25.18 (−56.7, 6.52)	−6.09 (−36.67, 24.48)	0.71 (−33.84, 35.07)	−29.3 (−61.28, 2.68)	−19.06 (−50.74, 12.89)	−6.69 (−36.76, 23.34)	14.04 (−26.26, 54.29)	−7.48 (−38.78, 23.73)	GCE	
3.69 (−25.01, 32.76)	5.67 (−21.45, 32.66)	4.35 (−18.43, 27.25)	−6.97 (−35.65, 21.79)	6.86 (−27.55, 40.93)	−15.76 (−44.66, 13.34)	3.28 (−24.4, 30.93)	10.08 (−21.92, 41.86)	−19.91 (−49.2, 9.32)	−9.65 (−38.69, 19.53)	2.71 (−24.23, 29.69)	23.46 (−14.78, 61.26)	1.91 (−26.54, 30.56)	9.42 (−22.2, 40.85)	GSE
6.33 (−19.22, 31.56)	8.35 (−15.32, 31.4)	7.05 (−11.39, 25.19)	−4.29 (−29.83, 20.79)	9.53 (−22.5, 40.7)	−13.06 (−38.88, 12.33)	5.94 (−18.37, 29.94)	12.75 (−16.2, 41.23)	−17.22 (−43.46, 8.57)	−6.95 (−32.99, 18.43)	5.39 (−18.22, 28.48)	26.08 (−9.68, 61.61)	4.63 (−20.7, 29.06)	12.11 (−16.65, 40.26)	2.73 (−22.99, 27.62)
−10.6 (−43.94, 22.68)	−8.58 (−40.54, 23.1)	−9.92 (−38.31, 18.36)	−21.28 (−54.46, 11.83)	−7.48 (−45.69, 30.82)	−30.01 (−63.36, 3.25)	−11.04 (−43.4, 21.26)	−4.23 (−40.32, 31.69)	−34.25 (−67.93, −0.75)	−23.92 (−57.43, 9.48)	−11.6 (−43.37, 20.13)	9.13 (−32.71, 50.89)	−12.34 (−45.39, 20.38)	−4.93 (−40.59, 31.14)	−14.31 (−47.51, 18.93)
9.29 (−22.8, 41.61)	11.29 (−19.6, 42.11)	9.98 (−17.01, 37.37)	−1.42 (−33.56, 31.14)	12.47 (−25.31, 50.12)	−10.17 (−42.67, 22.39)	8.82 (−22.28, 40.44)	15.67 (−19.47, 51)	−14.34 (−46.98, 18.67)	−4.06 (−36.71, 28.8)	8.3 (−22.53, 39.25)	29.01 (−11.82, 69.91)	7.54 (−24.28, 39.47)	15.05 (−20.05, 49.93)	5.6 (−26.68, 37.95)
−7.86 (−33.97, 18.42)	−5.85 (−29.89, 18.35)	−7.16 (−26.41, 12.23)	−18.54 (−44.49, 7.4)	−4.69 (−36.79, 27.41)	−27.25 (−53.53, −1.04)	−8.27 (−33.04, 16.69)	−1.46 (−30.9, 28.04)	−31.44 (−58.26, −4.69)	−21.19 (−47.51, 5.19)	−8.87 (−32.94, 15.21)	11.94 (−24.37, 48.25)	−9.55 (−35.24, 16.05)	−2.13 (−31.24, 26.87)	−11.57 (−37.58, 14.51)
−5.69 (−26.07, 14.9)	−3.66 (−21.58, 14.18)	−4.99 (−15.29, 5.5)	−16.34 (−36.63, 3.88)	−2.54 (−30.49, 25.16)	−25.1 (−45.65, −4.49)	−6.11 (−24.87, 12.89)	0.73 (−23.81, 25.27)	−29.25 (−50.43, −8.14)	−19.01 (−39.73, 1.76)	−6.68 (−24.46, 11.25)	14.12 (−18.4, 46.35)	−7.4 (−27.29, 12.41)	0.08 (−24.21, 24.08)	−9.35 (−29.71, 10.88)
−0.79 (−30.44, 29.05)	1.2 (−26.81, 29.24)	−0.11 (−23.9, 23.77)	−11.44 (−41.11, 18.26)	2.35 (−32.64, 37.4)	−20.24 (−49.76, 9.68)	−1.23 (−29.72, 27.32)	5.59 (−27, 38.32)	−24.38 (−54.55, 5.77)	−14.14 (−43.85, 15.75)	−1.79 (−29.65, 26.18)	18.96 (−19.61, 57.56)	−2.55 (−31.83, 26.8)	4.87 (−27.28, 37.46)	−4.51 (−33.83, 25.11)
1.54 (−27.16, 30.16)	3.51 (−23.67, 30.55)	2.17 (−20.45, 24.77)	−9.15 (−37.76, 19.28)	4.67 (−29.66, 38.66)	−17.96 (−46.61, 10.72)	1.08 (−26.61, 28.61)	7.9 (−23.87, 39.63)	−22.06 (−51.26, 6.98)	−11.8 (−40.78, 17.02)	0.5 (−26.38, 27.43)	21.33 (−16.8, 58.84)	−0.22 (−28.54, 27.83)	7.24 (−24.01, 38.47)	−2.2 (−30.85, 26.35)
1.57 (−27.35, 31)	3.58 (−23.91, 31.1)	2.29 (−20.96, 25.65)	−9.07 (−38.22, 20.05)	4.74 (−29.76, 39.44)	−17.82 (−47, 11.57)	1.21 (−26.78, 29.29)	8.04 (−24.11, 40.37)	−21.99 (−51.59, 7.88)	−11.71 (−41.06, 17.93)	0.61 (−26.67, 28.27)	21.38 (−17.04, 59.65)	−0.13 (−28.76, 28.72)	7.36 (−24.5, 39.39)	−2.08 (−31.07, 27.23)
−7.37 (−48.53, 33.84)	−5.4 (−45.52, 34.29)	−6.64 (−44.27, 30.38)	−18.02 (−59.44, 22.64)	−4.28 (−49.66, 40.75)	−26.75 (−68.14, 14.32)	−7.82 (−48.32, 32.42)	−1.01 (−44.55, 41.99)	−31.01 (−72.61, 10.53)	−20.66 (−62.43, 20.42)	−8.35 (−48.73, 31.38)	12.37 (−36.21, 60.24)	−9.07 (−50.19, 31.38)	−1.64 (−45.11, 41.45)	−11.05 (−52.38, 29.87)
7.47 (−24.59, 39.66)	9.48 (−21.15, 40.04)	8.15 (−18.83, 35.16)	−3.17 (−35.19, 28.77)	10.65 (−26.88, 47.76)	−11.95 (−44.32, 20.37)	7.07 (−23.87, 38.34)	13.86 (−21.08, 48.69)	−16.09 (−48.77, 16.27)	−5.84 (−38.13, 26.47)	6.46 (−23.94, 36.97)	27.25 (−13.81, 67.91)	5.76 (−26.1, 37.26)	13.23 (−21.54, 47.79)	3.81 (−28.41, 35.92)
−1.04 (−26.15, 24.06)	0.95 (−22.16, 23.85)	−0.35 (−18.31, 17.43)	−11.68 (−36.95, 13.32)	2.09 (−29.19, 33.18)	−20.47 (−45.71, 4.81)	−1.45 (−20.31, 17.35)	5.33 (−23.07, 33.85)	−24.57 (−50.38, 0.89)	−14.37 (−39.77, 10.9)	−2.03 (−25.19, 20.96)	18.73 (−16.78, 53.97)	−2.75 (−27.37, 21.84)	4.67 (−23.53, 32.7)	−4.69 (−29.79, 20.05)
0.15 (−29.78, 30.06)	2.17 (−26.01, 30.35)	0.86 (−23.41, 25.12)	−10.52 (−40.15, 19.21)	3.31 (−31.91, 38.53)	−19.28 (−49.32, 10.64)	−0.28 (−28.88, 28.62)	6.47 (−26.16, 39.49)	−23.41 (−53.89, 6.82)	−13.21 (−43.29, 17.12)	−0.84 (−28.95, 27.23)	19.93 (−19.02, 58.83)	−1.59 (−31.02, 28.06)	5.86 (−26.93, 38.58)	−3.55 (−33.14, 26.15)
7.65 (−24.27, 39.84)	9.63 (−20.64, 39.96)	8.37 (−18.36, 35.17)	−3 (−34.88, 28.93)	10.81 (−26.38, 47.93)	−11.76 (−43.92, 20.5)	7.26 (−23.76, 38.39)	14.06 (−20.83, 48.98)	−15.95 (−48.49, 16.49)	−5.69 (−37.89, 26.67)	6.65 (−23.7, 37.04)	27.42 (−13.03, 67.94)	5.91 (−25.7, 37.59)	13.36 (−21.29, 47.86)	4 (−27.9, 35.91)
5.32 (−23.14, 34.02)	7.3 (−19.36, 34.31)	6.02 (−16.46, 28.59)	−5.32 (−33.93, 22.92)	8.49 (−25.83, 42.59)	−14.12 (−42.9, 14.73)	4.89 (−22.47, 32.52)	11.67 (−19.77, 43.37)	−18.3 (−47.26, 10.89)	−8.04 (−36.63, 21.01)	4.31 (−22.55, 31.07)	25.12 (−12.9, 62.97)	3.6 (−24.75, 31.8)	11.03 (−20.03, 42.29)	1.63 (−26.78, 29.96)
8.17 (−22.02, 38.27)	10.15 (−18.67, 38.51)	8.88 (−15.91, 33.46)	−2.52 (−32.8, 27.75)	11.27 (−24.65, 46.96)	−11.26 (−41.71, 19.13)	7.77 (−21.6, 36.97)	14.54 (−18.77, 47.62)	−15.43 (−46.22, 15.22)	−5.16 (−35.88, 25.41)	7.14 (−21.48, 35.85)	27.91 (−11.48, 67.01)	6.43 (−23.68, 36.07)	13.91 (−19.14, 46.62)	4.47 (−25.69, 34.75)
GTE														
−16.93 (−47.24, 13.43)	HSE													
2.92 (−26.13, 32.46)	19.93 (−16.77, 56.57)	KRG												
−14.21 (−36.05, 8.28)	2.75 (−28.28, 33.94)	−17.09 (−47.18, 12.81)	Oli											
−12.02 (−26.89, 3.27)	4.93 (−21.3, 31.36)	−14.95 (−40.36, 10.13)	2.18 (−14.12, 18.52)	Pla										
−7.16 (−33.23, 19.28)	9.81 (−24.19, 43.9)	−10.08 (−43.21, 23.05)	7.03 (−19.97, 34.11)	4.86 (−16.52, 26.33)	PN									
−4.87 (−29.77, 20.35)	12.17 (−21, 45.15)	−7.81 (−40.25, 24.17)	9.35 (−16.42, 35.21)	7.16 (−13.01, 27.12)	2.35 (−27.23, 31.56)	Pom								
−4.73 (−30.07, 21.37)	12.23 (−21.29, 45.93)	−7.67 (−40.41, 25.08)	9.46 (−16.78, 35.8)	7.27 (−13.46, 28.19)	2.45 (−27.39, 32.35)	0.07 (−28.56, 29.16)	Pur							
−13.69 (−52.77, 25.05)	3.23 (−41.22, 47.52)	−16.64 (−60.55, 26.75)	0.47 (−39.18, 39.65)	−1.7 (−37.78, 33.96)	−6.55 (−48.44, 34.92)	−8.88 (−49.91, 31.82)	−9 (−50.52, 32.03)	RBAC						
1.12 (−27.78, 30.29)	18.13 (−18.03, 54.25)	−1.78 (−37.3, 33.43)	15.38 (−14.48, 45.02)	13.16 (−11.74, 37.96)	8.24 (−24.48, 41.02)	5.94 (−25.75, 37.78)	5.87 (−26.55, 38.15)	14.9 (−28.69, 58.58)	Res					
−7.4 (−28.13, 13.91)	9.54 (−20.36, 39.59)	−10.29 (−39.43, 18.55)	6.82 (−15.12, 28.53)	4.62 (−9.95, 19.08)	−0.24 (−26.2, 25.5)	−2.55 (−27.34, 22.23)	−2.65 (−27.95, 22.52)	6.35 (−32.08, 45.08)	−8.49 (−37.29, 20.15)	Sil				
−6.24 (−32.39, 20.55)	10.78 (−23.16, 44.89)	−9.07 (−42.45, 24.05)	7.99 (−19.24, 35.29)	5.81 (−15.97, 27.65)	0.95 (−29.63, 31.61)	−1.33 (−30.79, 28.17)	−1.49 (−31.66, 28.85)	7.58 (−34.01, 49.74)	−7.34 (−40.45, 25.87)	1.18 (−24.99, 27.5)	ST			
1.31 (−27.49, 30.37)	18.3 (−17.84, 54.33)	−1.63 (−37.06, 33.6)	15.5 (−14.14, 45.28)	13.34 (−11.37, 38)	8.49 (−24.44, 41.14)	6.16 (−25.71, 37.87)	6.11 (−26.35, 38.24)	15.05 (−28.36, 58.91)	0.15 (−34.95, 35.16)	8.66 (−19.81, 37.35)	7.46 (−25.47, 40.43)	TR		
−1.02 (−25.92, 24.17)	15.95 (−16.99, 49.02)	−3.91 (−36.22, 28.03)	13.16 (−12.59, 38.87)	10.99 (−8.99, 31.09)	6.11 (−23.26, 35.38)	3.84 (−24.56, 32.21)	3.7 (−25.34, 32.64)	12.7 (−27.9, 53.79)	−2.18 (−33.83, 29.62)	6.35 (−18.32, 31.13)	5.17 (−24.45, 34.91)	−2.36 (−34.08, 29.35)	Xan	
1.78 (−24.97, 28.94)	18.79 (−15.86, 53.39)	−1.07 (−35, 32.36)	15.96 (−11.67, 43.7)	13.82 (−8.56, 36.1)	8.96 (−22.01, 40.02)	6.68 (−23.36, 36.72)	6.53 (−24.11, 36.94)	15.5 (−26.41, 57.95)	0.69 (−32.99, 33.98)	9.19 (−17.54, 35.83)	7.99 (−23.42, 39.15)	0.5 (−32.93, 33.81)	2.83 (−27.23, 32.71)	ZM

**FIGURE 4:**
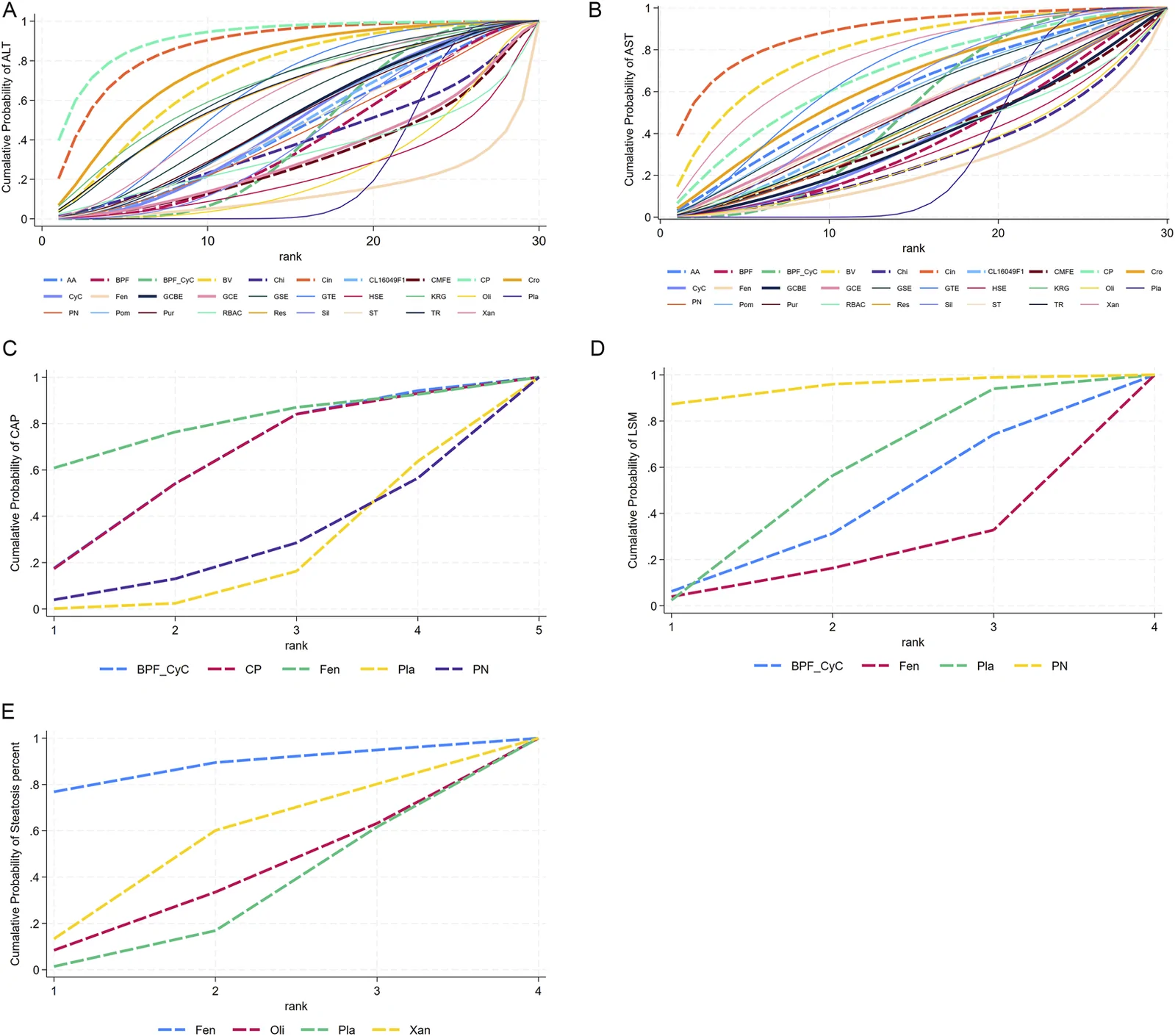
Cumulative probability ranking (SUCRA plot). **(A)** ALT. **(B)** AST. **(C)** CAP. **(D)** LSM. **(E)** Hepatic steatosis percentage.

#### 3.5.4 AST

The league table for AST is provided in Table 3. None of the pairwise differences reached statistical significance, as all 95% CrIs crossed zero (P > 0.05). The SUCRA ranking showed that Cin (SUCRA: 88.48%), BV (SUCRA: 80.42%), and Xanthigen (Xan) (SUCRA: 75.40%) had a higher probability of being top-ranked for AST reduction (Figure 4B and Supplementary Appendix 5).

**TABLE 3:** Network meta-analysis league table for AST.

AA														
−8.29 (−39.94, 23.25)	BPF													
−3.56 (−30.11, 22.75)	4.74 (−15.87, 25.36)	BPF_CyC												
9.15 (−24.28, 42.29)	17.43 (−13.69, 48.36)	12.68 (−13.27, 38.47)	BV											
−11.87 (−46.29, 22.56)	−3.6 (−35.85, 28.64)	−8.34 (−35.55, 19.06)	−21.05 (−54.93, 13)	Chi										
15.26 (−19.02, 49.35)	23.56 (−8.38, 55.61)	18.81 (−8.28, 46.03)	6.19 (−27.77, 39.93)	27.2 (−7.71, 61.92)	Cin									
−3.74 (−35.89, 28.54)	4.57 (−25.4, 34.5)	−0.15 (−24.64, 24.61)	−12.87 (−44.6, 19.27)	8.2 (−24.44, 41.13)	−19.06 (−51.74, 14.01)	CL16049F1								
−7.8 (−42.99, 27.32)	0.48 (−32.35, 33.49)	−4.22 (−32.35, 24.07)	−16.91 (−51.57, 17.83)	4.12 (−31.72, 39.71)	−23.02 (−58.76, 12.64)	−4.04 (−37.83, 29.52)	CMFE							
3.59 (−30.43, 37.8)	11.91 (−19.83, 43.87)	7.2 (−19.66, 34.14)	−5.47 (−38.96, 28.3)	15.51 (−19, 50.18)	−11.67 (−46.1, 22.95)	7.36 (−25.29, 39.73)	11.43 (−23.94, 47.04)	CP						
1.47 (−32.2, 34.93)	9.8 (−21.62, 41.07)	5.06 (−21.14, 31.19)	−7.66 (−40.51, 25.48)	13.36 (−20.78, 47.59)	−13.78 (−47.94, 20.22)	5.19 (−26.89, 37.21)	9.3 (−25.55, 44.04)	−2.2 (−36.43, 31.76)	Cro					
−7.29 (−39, 24.27)	1 (−21.83, 23.75)	−3.77 (−24.33, 16.91)	−16.43 (−47.31, 14.64)	4.63 (−27.57, 36.73)	−22.63 (−54.52, 9.35)	−3.53 (−33.55, 26.04)	0.5 (−32.52, 33.37)	−10.95 (−42.64, 20.88)	−8.78 (−39.94, 22.59)	CyC				
−14.08 (−48.59, 20.2)	−5.77 (−37.97, 26.61)	−10.52 (−37.81, 17.08)	−23.2 (−57.18, 11)	−2.18 (−37.2, 33.14)	−29.4 (−64.27, 5.65)	−10.36 (−43.32, 22.85)	−6.26 (−42.01, 29.74)	−17.71 (−52.66, 17)	−15.52 (−49.77, 18.56)	−6.76 (−39.01, 25.61)	Fen			
−8.12 (−41.82, 25.26)	0.21 (−31.28, 31.34)	−4.59 (−30.84, 21.62)	−17.23 (−50.3, 15.72)	3.76 (−30.52, 37.85)	−23.44 (−57.58, 10.62)	−4.43 (−36.63, 27.69)	−0.37 (−35.31, 34.53)	−11.75 (−45.8, 22.13)	−9.61 (−42.92, 23.72)	−0.88 (−32.25, 30.62)	5.98 (−28.43, 40.16)	GCBE		
−3.35 (−37.59, 30.91)	4.93 (−27.02, 37.02)	0.19 (−26.73, 27.33)	−12.53 (−46.33, 21.45)	8.52 (−26.29, 43.42)	−18.66 (−53.3, 16.35)	0.34 (−32.61, 33.11)	4.38 (−31.11, 40.09)	−6.94 (−41.42, 27.43)	−4.85 (−38.82, 29.16)	3.94 (−28.02, 36.03)	10.72 (−24.24, 45.65)	4.8 (−29.15, 38.89)	GCE	
−5.93 (−39.01, 27.13)	2.37 (−28.57, 33.24)	−2.38 (−28.07, 23.38)	−15.05 (−47.56, 17.58)	6.04 (−27.67, 39.57)	−21.19 (−54.5, 12.47)	−2.18 (−33.87, 29.31)	1.88 (−32.46, 36.38)	−9.57 (−43.26, 24.01)	−7.42 (−40.14, 25.47)	1.39 (−29.4, 32.28)	8.14 (−25.77, 42.17)	2.23 (−30.68, 35.14)	−2.54 (−36.17, 31)	GSE
2.68 (−26.44, 31.63)	11 (−15.66, 37.32)	6.22 (−13.93, 26.33)	−6.45 (−34.78, 22)	14.58 (−15.18, 44.08)	−12.62 (−42.21, 17.31)	6.43 (−20.98, 33.63)	10.47 (−20.19, 41.05)	−0.93 (−30.46, 28.28)	1.22 (−27.67, 29.92)	10.04 (−16.49, 36.38)	16.74 (−13.38, 46.44)	10.84 (−17.99, 39.56)	6.06 (−23.75, 35.54)	8.62 (−19.74, 36.68)
−10.27 (−44.23, 23.84)	−1.95 (−33.75, 29.93)	−6.7 (−33.54, 20.31)	−19.39 (−52.86, 14.47)	1.7 (−32.96, 36.16)	−25.53 (−60.02, 8.97)	−6.52 (−39.22, 26.06)	−2.47 (−37.76, 33.18)	−13.9 (−48.06, 20.59)	−11.73 (−45.48, 22.32)	−2.97 (−34.67, 29.06)	3.82 (−31.05, 38.71)	−2.1 (−35.82, 31.62)	−6.87 (−41.4, 27.82)	−4.35 (−37.67, 29.23)
−5.63 (−39.51, 28.42)	2.66 (−28.98, 34.51)	−2.05 (−28.66, 24.85)	−14.76 (−48.23, 19.01)	6.27 (−28.19, 41.04)	−20.87 (−55.16, 13.7)	−1.89 (−34.47, 30.72)	2.18 (−33.14, 37.79)	−9.27 (−43.47, 25.09)	−7.11 (−40.67, 26.67)	1.68 (−29.96, 33.64)	8.45 (−26.19, 43.34)	2.52 (−31.21, 36.55)	−2.23 (−36.73, 32.3)	0.31 (−32.94, 33.76)
−11.34 (−45.11, 22.26)	−2.99 (−34.3, 28.49)	−7.77 (−34.3, 18.77)	−20.47 (−53.57, 12.71)	0.56 (−33.87, 35.09)	−26.64 (−60.65, 7.91)	−7.6 (−39.85, 24.63)	−3.55 (−38.66, 31.66)	−14.98 (−49.04, 19.02)	−12.8 (−46.27, 20.66)	−4.04 (−35.39, 27.36)	2.74 (−31.78, 37.21)	−3.22 (−36.67, 30.34)	−7.98 (−42.26, 26.37)	−5.42 (−38.57, 27.77)
−8.58 (−32.41, 15.29)	−0.28 (−20.83, 20.32)	−5.03 (−16.4, 6.53)	−17.69 (−40.9, 5.63)	3.36 (−21.33, 27.99)	−23.87 (−48.47, 0.84)	−4.83 (−26.7, 16.91)	−0.78 (−26.57, 25.06)	−12.21 (−36.64, 12.18)	−10.07 (−33.58, 13.43)	−1.27 (−21.86, 19.34)	5.51 (−19.47, 30.28)	−0.43 (−23.9, 23.18)	−5.17 (−29.87, 19.35)	−2.63 (−25.67, 20.32)
−6.27 (−39.67, 27.17)	2 (−28.95, 33.02)	−2.74 (−28.51, 23.08)	−15.47 (−48.22, 17.47)	5.65 (−28.15, 39.58)	−21.57 (−55.11, 12.48)	−2.57 (−34.13, 29.18)	1.49 (−33.18, 36.04)	−9.93 (−43.52, 23.7)	−7.79 (−40.75, 25.51)	1.01 (−29.96, 31.99)	7.76 (−26.2, 41.84)	1.83 (−31.17, 35.13)	−2.92 (−36.49, 30.62)	−0.35 (−32.95, 32.12)
−0.91 (−34.16, 32.29)	7.36 (−23.76, 38.41)	2.59 (−23.41, 28.64)	−10.05 (−42.77, 22.73)	10.97 (−23.01, 44.85)	−16.2 (−49.95, 17.59)	2.76 (−29.08, 34.58)	6.81 (−27.84, 41.54)	−4.53 (−38.34, 29.07)	−2.45 (−35.55, 30.71)	6.37 (−24.65, 37.41)	13.09 (−20.79, 47.4)	7.22 (−25.72, 40.31)	2.44 (−31.32, 36.11)	5 (−27.84, 37.68)
−3.63 (−37.19, 29.85)	4.68 (−26.6, 35.78)	−0.1 (−26.15, 26.13)	−12.77 (−45.76, 20.35)	8.22 (−25.77, 42.58)	−18.93 (−52.85, 15.14)	0.09 (−31.99, 32.01)	4.19 (−30.69, 39.05)	−7.26 (−41.33, 26.5)	−5.08 (−38.5, 28.12)	3.67 (−27.5, 34.78)	10.41 (−23.64, 44.92)	4.51 (−28.65, 37.86)	−0.28 (−34.16, 33.48)	2.26 (−30.53, 35.15)
−8.43 (−45.53, 29.15)	−0.1 (−35.5, 35.35)	−4.87 (−35.69, 26.31)	−17.54 (−54.11, 19.45)	3.5 (−34.2, 41.39)	−23.71 (−61.26, 14.13)	−4.62 (−40.83, 31.24)	−0.63 (−39.15, 38.11)	−12.02 (−49.65, 25.75)	−9.85 (−46.74, 27.18)	−1.09 (−36.37, 34.39)	5.6 (−32.2, 43.95)	−0.28 (−37.46, 36.86)	−5.01 (−42.93, 32.81)	−2.52 (−39.06, 34.5)
−5.61 (−39.31, 27.65)	2.7 (−28.58, 33.73)	−2.05 (−28.33, 23.97)	−14.74 (−47.63, 18.23)	6.33 (−27.98, 40.13)	−20.87 (−54.89, 12.92)	−1.88 (−33.94, 29.97)	2.19 (−32.59, 37.03)	−9.22 (−43.09, 24.38)	−7.11 (−40.36, 26.09)	1.68 (−29.79, 32.78)	8.49 (−25.68, 42.43)	2.54 (−30.89, 35.73)	−2.23 (−36.3, 31.46)	0.35 (−32.77, 32.9)
−0.78 (−30.02, 28.26)	7.49 (−18.96, 34.04)	2.75 (−17.38, 23.08)	−9.9 (−38.32, 18.84)	11.1 (−18.51, 40.88)	−16.09 (−45.56, 13.78)	2.93 (−18.76, 24.74)	7.01 (−23.61, 37.75)	−4.41 (−33.87, 25.25)	−2.23 (−31.06, 26.77)	6.54 (−19.93, 33.01)	13.3 (−16.75, 43.12)	7.36 (−21.37, 36.31)	2.54 (−26.94, 32.39)	5.16 (−23.23, 33.53)
−3.13 (−36.89, 30.25)	5.13 (−25.88, 36.38)	0.39 (−25.58, 26.45)	−12.29 (−45.01, 20.72)	8.75 (−25.16, 42.98)	−18.44 (−52.39, 15.77)	0.59 (−31.4, 32.35)	4.6 (−30.12, 39.69)	−6.76 (−40.58, 26.83)	−4.61 (−37.81, 28.66)	4.13 (−26.89, 35.21)	10.87 (−23.29, 45.03)	4.96 (−28.13, 38.55)	0.18 (−33.7, 34.2)	2.73 (−29.93, 35.53)
−5.23 (−38.89, 28.41)	3.08 (−28.14, 34.53)	−1.68 (−27.76, 24.71)	−14.32 (−47.26, 18.66)	6.7 (−27.27, 40.84)	−20.51 (−54.41, 13.64)	−1.46 (−33.74, 30.36)	2.58 (−32.27, 37.49)	−8.82 (−42.74, 25.04)	−6.7 (−39.93, 26.47)	2.09 (−29.23, 33.45)	8.84 (−25.37, 43.05)	2.93 (−30.24, 36.3)	−1.82 (−35.8, 32.11)	0.71 (−32.19, 33.61)
6.44 (−26.74, 39.45)	14.7 (−16.08, 45.6)	9.99 (−15.84, 35.57)	−2.67 (−35.3, 29.97)	18.28 (−15.36, 52.26)	−8.86 (−42.62, 25.04)	10.17 (−21.6, 41.75)	14.26 (−20.22, 48.65)	2.81 (−30.82, 36.03)	4.95 (−27.93, 38.09)	13.7 (−17.12, 44.58)	20.52 (−13.45, 54.27)	14.58 (−18.27, 47.66)	9.8 (−23.75, 43.47)	12.42 (−20.29, 44.73)
−1.25 (−34.92, 32.01)	7 (−24.41, 38.43)	2.25 (−23.99, 28.54)	−10.39 (−43.31, 22.49)	10.62 (−23.24, 44.66)	−16.56 (−50.63, 17.53)	2.46 (−29.86, 34.58)	6.49 (−28.27, 41.31)	−4.89 (−38.89, 28.77)	−2.74 (−36.11, 30.38)	6.04 (−25.29, 37.24)	12.8 (−21.45, 46.99)	6.82 (−26.35, 40.06)	2.13 (−31.87, 36.2)	4.61 (−28.2, 37.38)
GTE														
−12.95 (−42.25, 16.56)	HSE													
−8.33 (−37.57, 21.38)	4.62 (−29.76, 39.17)	KRG												
−14.04 (−43.01, 15.06)	−1.11 (−35.16, 33)	−5.73 (−39.81, 27.96)	Oli											
−11.26 (−27.83, 5.33)	1.7 (−22.65, 25.95)	−2.96 (−27.35, 21.23)	2.77 (−21.19, 26.66)	Pla										
−9.02 (−37.38, 19.63)	3.94 (−29.53, 37.44)	−0.72 (−34.43, 32.85)	5.03 (−28.06, 38.23)	2.27 (−20.86, 25.45)	PN									
−3.64 (−32.08, 25.17)	9.31 (−24.23, 42.93)	4.63 (−28.95, 38.27)	10.39 (−22.84, 43.62)	7.64 (−15.52, 30.88)	5.34 (−27.31, 38.09)	Pom								
−6.32 (−34.96, 22.46)	6.61 (−27.31, 40.28)	2.01 (−31.82, 35.62)	7.71 (−25.78, 40.89)	4.96 (−18.43, 28.34)	2.71 (−30.54, 35.48)	−2.67 (−35.77, 30.38)	Pur							
−11.09 (−44.03, 22.21)	1.83 (−35.86, 39.58)	−2.78 (−40.5, 34.88)	2.87 (−34.18, 40.35)	0.13 (−28.46, 29.01)	−2.11 (−39.09, 34.63)	−7.49 (−44.31, 29.8)	−4.78 (−41.9, 32.48)	RBAC						
−8.32 (−36.89, 20.28)	4.6 (−29.1, 38.3)	−0.03 (−33.94, 33.61)	5.75 (−27.68, 39.03)	2.95 (−20.68, 26.3)	0.73 (−32.36, 33.31)	−4.61 (−37.89, 28.11)	−1.98 (−35.28, 30.95)	2.85 (−34.56, 39.74)	Res					
−3.49 (−26.84, 20.19)	9.46 (−19.7, 38.91)	4.81 (−24.71, 34.14)	10.51 (−18.38, 39.81)	7.79 (−8.81, 24.45)	5.5 (−22.93, 34.2)	0.16 (−28.53, 28.66)	2.83 (−25.68, 31.65)	7.6 (−25.52, 40.89)	4.85 (−23.79, 33.8)	Sil				
−5.87 (−34.34, 22.75)	7.08 (−26.61, 40.66)	2.46 (−31.46, 36.1)	8.19 (−25.37, 41.82)	5.39 (−17.99, 28.79)	3.16 (−29.91, 36.02)	−2.21 (−35.22, 30.92)	0.43 (−32.77, 33.81)	5.21 (−31.76, 42.16)	2.45 (−30.76, 35.69)	−2.37 (−31.18, 26.25)	ST			
−7.91 (−36.43, 20.91)	5.03 (−28.67, 38.63)	0.4 (−33.52, 34.14)	6.08 (−27.22, 39.78)	3.36 (−20.1, 26.97)	1.09 (−31.81, 34.11)	−4.26 (−37.37, 28.76)	−1.58 (−34.8, 31.63)	3.18 (−34, 40.37)	0.43 (−32.83, 33.71)	−4.44 (−33.34, 24.41)	−2 (−35.43, 31.06)	TR		
3.75 (−24.55, 32.29)	16.68 (−16.62, 49.91)	12.06 (−21.44, 45.54)	17.73 (−15.42, 50.9)	15 (−8.1, 37.98)	12.71 (−19.98, 45.32)	7.4 (−25.43, 39.98)	10.07 (−22.9, 42.93)	14.86 (−21.97, 51.39)	12.04 (−20.65, 44.83)	7.2 (−21.18, 35.43)	9.57 (−23.17, 42.38)	11.71 (−21.34, 44.41)	Xan	
−3.97 (−32.77, 24.81)	8.96 (−24.65, 42.82)	4.33 (−29.52, 37.93)	10.04 (−23.47, 43.64)	7.25 (−16.34, 30.77)	5.03 (−28.17, 38)	−0.32 (−33.39, 32.65)	2.36 (−30.92, 35.64)	7.17 (−30.08, 44.19)	4.33 (−28.74, 37.54)	−0.48 (−29.65, 28.35)	1.93 (−31.32, 34.81)	3.9 (−29.43, 37.27)	−7.72 (−40.58, 25.01)	ZM

#### 3.5.5 LSM

The league table for LSM is shown in Table 4. No intervention showed a statistically significant advantage over others, as all 95% CrIs crossed zero (P > 0.05). According to the SUCRA ranking, Pur (SUCRA: 94.16%) had a higher probability of being top-ranked for reducing LSM levels (Figure 4C and Supplementary Appendix 5).

**TABLE 4:** Network meta-analysis league table for LSM.

BPF + CyC	​	​	​
−0.49 (−2.43, 1.45)	Fen	​	​
0.2 (−0.96, 1.36)	0.7 (−0.87, 2.28)	Pla	​
1.03 (−0.54, 2.6)	1.53 (−0.35, 3.41)	0.83 (−0.24, 1.89)	Pur

#### 3.5.6 CAP

The league table for CAP is presented in Table 5. None of the pairwise differences reached statistical significance, as all 95% CrIs exceeded zero (P > 0.05). Based on the SUCRA ranking, Fen (SUCRA: 79.07%), BFC + CyC (SUCRA: 62.57%), and CP (SUCRA: 61.99%) had a higher probability of being top-ranked for reducing CAP values (Figure 4D and Supplementary Appendix 5).

**TABLE 5:** Network meta-analysis league table for CAP.

BPF + CyC	​	​	​	​
−0.16 (−43.43, 43.87)	CP	​	​	​
14.48 (−42.29, 70.84)	14.48 (−44.88, 73.84)	Fen	​	​
−15.53 (−42.58, 12.44)	−15.39 (−49.51, 18.81)	−29.91 (−79.15, 20.08)	Pla	​
−15.28 (−59.69, 29.73)	−15.18 (−64.23, 33.87)	−29.7 (−89.58, 30.78)	0.25 (−34.88, 35.44)	PN

#### 3.5.7 Hepatic steatosis percentage

The league table for hepatic steatosis percentage is provided in Table 6, demonstrating that none of the pairwise differences was statistically significant, as all 95% CrIs crossed zero (P > 0.05). The SUCRA ranking showed that Fen (SUCRA: 86.97%) had a higher probability of being top-ranked for lowering hepatic steatosis percentage (Figure 4E and Supplementary Appendix 5).

**TABLE 6:** Network meta-analysis league table for hepatic steatosis percentage.

Fen	​	​	​
−22.45 (−69.12, 24.22)	Oli	​	​
−23.57 (−59.74, 12.84)	−1.1 (−30.87, 28.88)	Pla	​
−18.62 (−64.86, 27.82)	3.85 (−37.99, 45.77)	4.97 (−24.75, 34.37)	Xan

#### 3.5.8 Convergence diagnosis

Convergence diagnostics indicated satisfactory convergence for all NMA models (ALT, AST, CAP, LSM, and hepatic steatosis percentage). Trace plots showed favorable mixing and stable trajectories of the Markov chains over iterations (Supplementary Appendix 6), confirming that the posterior estimates were adequately stable.

#### 3.5.9 Heterogeneity analysis

Heterogeneity was assessed using the I^2^ statistic. Substantial heterogeneity was observed for ALT, with pairwise I^2^ = 97.38% and overall I^2^ = 98.46%. Similarly, AST showed considerable heterogeneity, with pairwise I^2^ = 98.05% and overall I^2^ = 98.57% (Supplementary Appendix 7).

#### 3.5.10 Exploration of heterogeneity sources

##### 3.5.10.1 Network meta-regression analysis

Network meta-regression analyses were performed to investigate whether treatment duration and dosage contributed to the observed heterogeneity. Neither duration nor dosage significantly moderated the treatment effects on ALT or AST. For ALT, each 4-week increase in duration was associated with a change in MD of −0.21 (95% CrI: −12.96 to 12.55), and each doubling of dosage corresponded to a change in MD of −8.63 (95% CrI: −32.33 to 18.32); neither was statistically significant. For AST, the corresponding changes in MD were −14.76 (95% CrI: −50.15 to 21.60) per 4-week extension and −31.88 (95% CrI: −53.90 to 1.15) per dose doubling, also without statistical significance. These results suggested a trend toward greater liver enzyme reductions with longer treatment or higher dosage. However, the current evidence is insufficient to establish a clear duration- or dose-response relationship. Details are available in Supplementary Appendix 8.

##### 3.5.10.2 Subgroup analysis

To further explore whether the presence of structured lifestyle interventions influences the therapeutic effects of plant extracts and leads to heterogeneity, we performed an exploratory subgroup analysis stratified by the presence or absence of such interventions. Details are available in Supplementary Appendix 7. For ALT, the pairwise I^2^ and overall I^2^ in the subgroup with structured lifestyle interventions were 50.24% and 51.04%, respectively, whereas the corresponding values in the subgroup without structured lifestyle interventions or without such reports were both 0%. For AST, the pairwise I^2^ and overall I^2^ in the structured lifestyle intervention subgroup were 52.97% and 53.00%, respectively, while in the subgroup without structured lifestyle interventions or without such reports, the pairwise I^2^ was 100% and the overall I^2^ was 0%. Overall, stratification reduced heterogeneity estimates in some subgroups, suggesting that structured lifestyle interventions may be one potential factor influencing differences in treatment effects. However, given the limited number of studies and sparse network structure in certain subgroups, the corresponding heterogeneity estimates remain uncertain. In addition, the intervention rankings for ALT and AST within each lifestyle subgroup were generally consistent with those from the overall analysis (Supplementary Appendix 9), indicating that the main conclusions were reasonably robust across different lifestyle intervention contexts.

Additional subgroup analyses were performed according to plant source (single-component extracts, medicine-food homology extracts, and compound preparations). The efficacy rankings within each subgroup were highly consistent with those from the overall analysis for ALT and AST (Supplementary Appendix 10). However, heterogeneity remained high after stratification Supplementary Appendix 1), suggesting that plant source was not a major driver of the observed heterogeneity.

### 3.6 CINeMA assessment

The certainty of evidence for each comparison was assessed using the CINeMA framework. Overall, the certainty of evidence was limited across outcomes. For ALT reduction, only the comparison between CP and placebo was rated as being of moderate certainty, whereas all other comparisons were judged as being of low or very low certainty. For AST reduction, all comparisons were rated as being of low or very low certainty. The main reasons for downgrading were imprecision, within-study bias, and heterogeneity. CINeMA assessment is detailed in Supplementary Appendix 11,12.

### 3.7 Publication bias

Publication bias was assessed through comparison-adjusted funnel plots. In the funnel plots for ALT and AST, the points were distributed symmetrically, indicating no evidence of publication bias (Figure 5).

**FIGURE 5:**
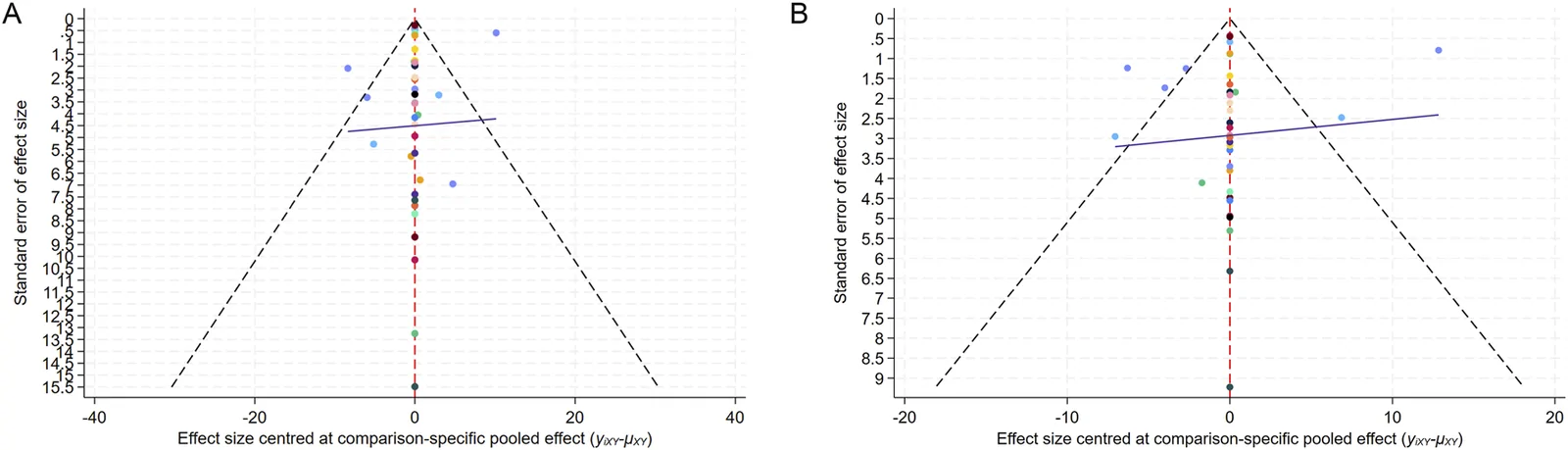
Publication bias. **(A)** ALT. **(B)** AST.

## 4 Discussion

This study compared the effects of 28 types of plant extracts across 5 liver-related outcomes (ALT, AST, CAP, LSM, and hepatic steatosis percentage) among patients with MASLD. A total of 32 RCTs involving 2,053 participants were included. The analysis suggested that certain plant extracts showed potential advantages for specific liver-related outcomes. Among liver biochemical indicators, CP (Curcuvail®) had the highest probability of ranking first for ALT reduction, whereas Cin had the highest probability of ranking first for AST reduction. Fen showed a favorable ranking for lowering CAP values and hepatic steatosis percentage, while Pur ranked highest for reducing LSM. Nevertheless, these SUCRA-based rankings should be interpreted with caution and in conjunction with the magnitude and precision of the effect estimates, as well as the certainty of the evidence. In this study, most comparisons were supported by low or very low certainty evidence according to CINeMA, mainly due to imprecision, heterogeneity, and methodological limitations. Therefore, a higher ranking probability does not necessarily indicate clinically meaningful superiority, particularly when confidence intervals are wide or substantially overlap across interventions. Consistent with the scoping review by Handu et al. (Handu et al., 2025), our NMA confirmed that curcumin, alongside silymarin, resveratrol, coffee, green tea, and berberine, were among the most frequently investigated plant-derived compounds in MASLD. Moreover, although the recent NMA by Sairaj et al. (Sairaj et al., 2026) emphasized improvements in systemic metabolic parameters following herbal interventions, our analysis mainly focused on hepatic-specific outcomes and revealed that the relative performance of plant extracts varies across different domains. This discrepancy suggests that strong performance on inflammatory or metabolic markers does not necessarily translate into comparable improvements in liver-specific endpoints such as transaminases, steatosis, or stiffness.

Among the interventions assessed, CP merits particular attention, as our findings diverge from earlier evidence syntheses. CP (Patel et al., 2025) demonstrated a favorable probability of reducing ALT levels, differing from the findings of Liu et al. (Liu et al., 2024), who reported limited efficacy of curcumin in MASLD. Unlike conventional CPs, CP (CurcuVail®) employs an optimized formulation that may enhance curcumin bioavailability, potentially augmenting its therapeutic effects. Moreover, our analysis included a recent clinical trial evaluating CP published in 2023 (Patel et al., 2025), which was not included in previous evidence syntheses. Therefore, the favorable results observed in this study may reflect the contribution of improved formulation and updated clinical evidence rather than a contradiction with previous reports. Supporting this interpretation, a recent randomized, double-blind, placebo-controlled trial of phospholipid curcumin (Meriva) reported improvements in liver histological outcomes among patients with nonalcoholic steatohepatitis (Musso et al., 2025). Collectively, these findings suggest that formulation optimization may enhance the clinical performance of curcumin-based interventions. However, histological outcomes were not assessed in this NMA; accordingly, whether such advantages extend to broader hepatic outcomes in MASLD requires further investigation.

Beyond curcumin-specific formulations, combination plant-derived interventions were also potentially effective; BPF + CyC demonstrated a higher probability of reducing AST than CyC alone, suggesting that combination therapy may provide additional benefits over single-component CPs. Preclinical evidence suggests that BPF and cynaropicrin may exert complementary metabolic effects, with BPF potentially enhancing β-oxidation and cynaropicrin mitigating inflammatory and oxidative stress responses (Mirarchi et al., 2022; Lee et al., 2021). Although these mechanisms provide a plausible explanation for the observed findings, whether combination therapy confers clinically meaningful advantages over single-component interventions remains to be confirmed in head-to-head RCTs in the future.

While the above findings highlight the potential importance of formulation optimization and multi-component strategies, the therapeutic effects of plant extracts may also arise from their intrinsic metabolic regulation. Cin also exhibited favorable effects on liver biochemical parameters, particularly AST. An experimental study has suggested that Cin-derived bioactive compounds may improve insulin sensitivity, which could partly account for the observed improvements in liver enzymes, given the central role of insulin resistance in MASLD progression (Cao et al., 2007).

Compared with previous meta-analyses that primarily evaluated biochemical indicators (e.g., ALT and AST), this study additionally assessed non-invasive liver-related surrogate markers (e.g., CAP, LSM, and hepatic steatosis percentage), thereby providing a broader assessment of hepatic structural changes in MASLD. Fen demonstrated favorable effects on CAP and hepatic steatosis percentage. Experimental evidence suggests that its bioactive constituents, including diosgenin, 4-hydroxyisoleucine, and dietary fiber, may contribute to the regulation of lipid metabolism (Fuller and Stephens, 2015), providing a possible explanation for these findings. For outcomes related to liver stiffness, Pur ranked highest for reducing LSM. Experimental studies have suggested that Pur-derived bioactive components may exert anti-inflammatory effects through pathways such as NF-κB signaling (Song et al., 2025; Li et al., 2025), which may partly explain the observed association. Nevertheless, whether these biological effects translate into clinically meaningful improvements remains uncertain, as the current evidence is derived from a limited number of RCTs.

Despite these promising signals, caution is warranted when interpreting their clinical significance. From a clinical perspective, the current evidence does not support the use of plant extracts as independent therapeutic agents for MASLD. Although several interventions, including novel CPs, Cin, Fen, and Pur, showed favorable probability rankings based on SUCRA values, these findings should be viewed as hypothesis-generating rather than definitive proof of clinical efficacy. In particular, improvements in surrogate markers such as liver enzymes, CAP, or LSM do not necessarily translate into meaningful reductions in long-term outcomes, including progression to advanced fibrosis, cirrhosis, hepatocellular carcinoma, or liver-related mortality (Tacke et al., 2024). Therefore, plant extracts should not replace established interventions, particularly lifestyle modification incorporating dietary improvement and physical activity, which remains the cornerstone of MASLD management.

Considerable heterogeneity was observed for some outcomes, which may stem from differences in patient characteristics, plant extract types, intervention measures, treatment duration, dosage, and concomitant lifestyle management across included trials. To explore possible sources of heterogeneity, network meta-regression analyses were performed to assess whether treatment dosage and duration influenced therapeutic effects. Neither dosage nor duration showed a statistically significant association with changes in ALT or AST, suggesting that intervention intensity alone may not fully explain the observed heterogeneity. Given the significant role of lifestyle modification in MASLD management, subgroup analyses were conducted according to the presence or absence of structured lifestyle interventions. In studies with structured lifestyle interventions, heterogeneity decreased from 98% to approximately 50%, implying that differences in lifestyle management may partly lead to variability in treatment effects. However, this finding should be interpreted with caution, as lifestyle interventions varied in dietary patterns, exercise regimens, and adherence strategies, and potential interactions between lifestyle factors and plant extracts cannot be excluded. Therefore, structured lifestyle intervention should not be considered the sole explanation for heterogeneity. Notably, heterogeneity in the subgroup without structured lifestyle intervention decreased from 100% to 0%. This apparent reduction should be interpreted with caution, as it may partly reflect the limited number of studies and reduced statistical power after subgroup analysis rather than true homogeneity among studies. In addition, subgroup analyses based on plant origin did not substantially reduce heterogeneity, suggesting that botanical source alone may not adequately explain differences in treatment effects. Other factors, including active compound composition, extraction methods, bioavailability, formulation characteristics, and baseline metabolic status, may also contribute to the observed variability.

Nevertheless, the absence of statistically significant superiority over placebo does not necessarily indicate that the fundamental therapeutic concept of plant extracts is invalid. Instead, it highlights several unresolved challenges in this field. The included trials varied substantially in extract formulations, bioactive compound concentrations, dosages, treatment durations, and patient characteristics, which may have diluted potential treatment effects. Moreover, many available studies had a small sample size and primarily focused on short-term biochemical or imaging surrogates rather than clinically meaningful endpoints. Research in the future should prioritize adequately powered RCTs with standardized plant extract formulations, rigorous control of lifestyle-related confounding, well-characterized patient populations, and clinically meaningful liver-specific outcomes, including validated histological endpoints where feasible. Such studies are essential to determine whether specific plant extracts can serve as effective adjuncts to lifestyle modification in carefully selected patients with MASLD.

This study systematically assessed the efficacy of multiple plant extracts through an NMA. However, several methodological limitations and sources of clinical uncertainty should be acknowledged. First, significant statistical heterogeneity was observed for ALT and AST, which reduces confidence in the pooled estimates and limits the interpretability of the treatment rankings. Second, the number of included studies assessing emerging interventions (e.g., Cin and Pur) and key non-invasive histological outcomes (e.g., CAP, LSM, and hepatic steatosis percentage) is small, with a limited sample size, constraining the robustness of the network model. Third, the included studies were predominantly performed in Iran, other Asian countries, and Mediterranean regions, raising concerns regarding the external validity and generalizability of our findings. MASLD is a highly heterogeneous metabolic disorder influenced by ethnicity, dietary patterns, genetic susceptibility, environmental exposures, and the distribution of metabolic comorbidities. Populations from different regions may differ substantially in the prevalence rates of obesity, insulin resistance, type 2 diabetes, dyslipidemia, and other metabolic abnormalities, which may influence both disease severity and the response to plant-based interventions. In addition, local dietary habits and gut microbiota profiles may affect the metabolism, absorption, and biological activity of plant-derived compounds, potentially modifying their therapeutic effects. Therefore, although the included studies provide valuable evidence regarding the potential benefits of plant extracts, the extent to which these findings can be generalized to MASLD populations with different ethnic backgrounds, lifestyle characteristics, and metabolic profiles remains uncertain. Fourth, the intervention durations varied widely across studies (from 4 weeks to 12 months), and long-term follow-up data on cirrhosis, liver cancer, and all-cause mortality were generally lacking. Finally, the standardization of active ingredients in plant extracts was insufficient, and significant differences in bioavailability among different preparations existed, making it difficult to completely normalize dose-effect relationships. Accordingly, in the absence of robust evidence, whether the short-term improvements observed with plant extracts can translate into long-term substantive clinical benefit remains highly uncertain, and large-scale, standardized, rigorously designed trials are needed to validate these findings in the future.

## 5 Conclusion

This NMA systematically assessed the comparative efficacy of 28 categories of plant extracts for MASLD. Across most comparisons, no statistically significant differences were detected between plant extracts and placebo, and the comparative evidence remained highly uncertain. Although several plant extracts, including CP, Cin, Fen, and Pur, showed favorable probability rankings for specific liver-related outcomes, these findings were based on limited evidence and should not be interpreted as establishing clinically meaningful superiority. Consequently, current evidence does not support the use of plant extracts as standalone therapeutic interventions for MASLD in the absence of concomitant lifestyle modification. In the future, adequately powered, methodologically rigorous RCTs are needed to elucidate whether specific plant extracts may serve as effective adjuncts to lifestyle intervention in this population.

## Data Availability

The original contributions presented in the study are included in the article/Supplementary Material, further inquiries can be directed to the corresponding author.
